# Lower Urinary Tract Symptoms and Sexual Dysfunction in Male: A Systematic Review and Meta-Analysis

**DOI:** 10.3389/fmed.2021.653510

**Published:** 2021-05-28

**Authors:** Guoda Song, Min Wang, Bingliang Chen, Gongwei Long, Hao Li, Rui Li, Zhuo Liu, Chao Wei, Tao Wang, Shaogang Wang, Jihong Liu, Yucong Zhang, Xiaming Liu

**Affiliations:** ^1^Department of Urology, Tongji Hospital, Tongji Medical College, Huazhong University of Science and Technology, Wuhan, China; ^2^Second Clinical College, Tongji Medical College, Huazhong University of Science and Technology, Wuhan, China; ^3^Department of Emergency and Intensive Care Unit, Tongji Hospital, Tongji Medical College, Huazhong University of Science and Technology, Wuhan, China; ^4^Department of Geriatric, Tongji Hospital, Tongji Medical College, Huazhong University of Science and Technology, Wuhan, China

**Keywords:** lower urinary tract symptoms, meta-analysis, sexual dysfunction, systematic review, erectile dysfunction

## Abstract

**Background:** An association between lower urinary tract symptoms (LUTS) and risk of sexual dysfunction in male remains controversial in recent decades.

**Materials and Methods:** PubMed and Web of Science were searched up to October 28, 2020, for articles reporting the prevalence of sexual dysfunction in men with LUTS. The main outcomes were results from sexual dysfunction assessments. Pooled odds ratio (OR) and weighted mean difference (WMD) with 95% confidence interval (CI) were calculated. The quality assessment of the included studies was performed by using The Newcastle-Ottawa Scale (NOS) or JBI Meta-Analysis of Statistics Assessment and Review Instrument (JBI-MAStARI).

**Results:** A total of 24 full-manuscript papers met the inclusion criteria. The pooled OR for 21 studies suggested that patients with severer LUTS had a higher risk of sexual dysfunction (OR = 3.31, 95% CI: 2.43 to 4.49, *p* < 0.001, *I*^2^ = 90%). A significant decrease in scores of assessment tools for sexual dysfunction was observed in the patients with higher severity of LUTS compared with those patients with lower severity (WMD = −5.49, 95%CI: −7.25 to −3.27, P < 0.001, *I*^2^ = 96%). Similar outcomes were also found in subgroup analyses. In a detailed analysis of specific sexual function domains, the severity of LUTS was associated with erectile dysfunction, intercourse satisfaction, and overall satisfaction, except for sexual desire.

**Conclusion:** The study demonstrates an association between exposure of lower urinary tract symptoms and risk of sexual dysfunction in male. Assessment of sexual function is necessary for patients with lower urinary tract symptoms.

**Systematic Review Registration:** http://www.crd.york.ac.uk/prospero, identifier: CRD42020208747.

## Introduction

Lower urinary tract symptoms (LUTS) are common in elderly men and the prevalence increases with age according to the epidemiological studies (1). LUTS include storage, voiding, and post micturition symptoms according to the International Continence Society (ICS) (2). Storage symptoms comprise increased daytime frequency, nocturia, urgency, and urinary incontinence. The voiding symptoms include slow stream, splitting or spraying of the urine stream, intermittent stream, hesitancy, straining, and terminal dribble. Post micturition symptoms refer to feeling of incomplete emptying and post micturition dribble (3, 4). These symptoms may be associated with structural and functional abnormalities of the urinary tract and surrounding tissues such as prostate, bladder or non-urological conditions (e.g., nocturia) (5). Nocturia and urgency are the most prevalent and bothersome symptoms. These symptoms strongly affected the quality of life (6).

Sexual dysfunction is defined as difficulty experienced by an individual or a couple during any stage of a normal sexual activity which results in misery and strained interpersonal relationship. Sexual dysfunction includes sexual desire disorders, sexual arousal disorders, orgasmic disorders, and sexual pain disorders. Erectile dysfunction belongs to sexual arousal disorder (7, 8). Sexual dysfunction might have a significant impact on the quality of life of patients and their partners (9, 10).

Some epidemiological studies have shown that there is an association between exposure of LUTS and risk of sexual dysfunction (11, 12). Understanding the prevalence of sexual dysfunction in men with LUTS will help the clinicians to better screen the high-risk population of sexual dysfunction and provide early intervention accordingly. Therefore, this meta-analysis was conducted to evaluate the association between LUTS and sexual dysfunction in men based on published studies.

## Materials and Methods

This work was executed in accordance with the Preferred Reporting Items for Systemic Reviews and Meta-analysis (PRISMA) guidelines. According to PRISMA guideline, in current study, the populations were adult male, the exposures were individuals with LUTS or higher severity of LUTS, the comparators were individuals without LUTS or with lower severity of LUTS, the outcomes were results of sexual dysfunction assessment. We have registered this meta-analysis in PROSPERO (CRD42020208747; www.crd.york.ac.uk/prospero).

### Search Strategy

Systematic literature search was conducted by searching the online databases including PubMed and Web of Science. Relevant studies, which assessed the association between LUTS and sexual dysfunction and were published up to October 28, 2020, were screened. The following search terms were used: “CP/CPPS,” “chronic prostatitis,” “chronic pelvic pain syndrome,” “lower urinary tract symptoms,” “LUTS,” “National Institutes of Health—Chronic Prostatitis Symptom Index,” “NIH-CPSI,” “IPSS,” “erectile dysfunction,” “erectile disorder,” “sexual dysfunction,” “ED,” “IIEF,” “international index of erectile function,” “sexual desire disorders,” “sexual arousal disorders,” “orgasmic disorders,” “sexual pain disorders,” “hypoactive sexual desire disorder,” “sexual aversion disorder,” “premature ejaculation” and “dyspareunia.” An English language restriction was implied. The references of included articles were also hand-searched to obtain additional studies.

### Selection Criteria

Included studies should meet the following criteria: (1) Studies reported the association between LUTS and sexual dysfunction. LUTS refer to a group of clinical symptoms involving the bladder, urinary sphincter, urethra and the prostate in men, which included increased frequency of urination, increased urgency of urination, urge incontinence, excessive passage of urine at night, poor stream, hesitancy, terminal dribbling, incomplete voiding, urinary retention, overflow incontinence and episodes of near retention. Sexual dysfunction included sexual desire disorders, sexual arousal disorders, erectile dysfunction, premature ejaculation, orgasm disorders, sexual pain disorders and post-orgasmic diseases; (2) Studies reported the prevalence or the number of sexual dysfunction patients, or the average scores with standard deviation of assessment tools for sexual function; (3) Clinical studies were performed with adult males and published in English. The exclusion criteria were as follows: (1) The study type was a review, case report, abstract of conference, comment or editorial. (2) Study without appropriate comparator. (3) Study reported incomplete data of outcomes, such as the lack of standard deviation of sexual function assessment score. (4) The determination of LUTS and ED didn't base on appropriate clinic diagnosis or relevant questionnaires. The questionnaires for LUTS assessment included International Prostate Symptom Score (IPSS) questionnaire, Danish Prostatic Symptom Score (DAN-PSS-1) questionnaire and The National Institutes of Health Chronic Prostatitis Symptom Index (NIH-CPSI). The questionnaires for sexual function assessment included International Index of Erectile Function (IIEF), the Brief Male Sexual Function Inventory (BSFI), Danish Prostatic Symptom Score (DAN-PSS-sex) and Epstein Inventory. The details of these questionnaires are provided in the Supplementary Material; (5) Patients with prostate cancer and those who have planned benign prostatic hyperplasia (BPH) surgery in a prospective study.

### Study Screening and Data Extraction

The titles and abstracts were screened independently by two authors, and then the full texts of the relevant studies were reviewed later. The reference lists of relevant articles were hand-searched. Two authors independently extracted and cross-checked the following data: first author, publication year, nation, age, study type, sample size, criteria of LUTS severity categories, assessment tools for LUTS and sexual function, and diagnosis criteria of sexual dysfunction. Any disagreements were discussed by the two authors or sought help from the third author.

### Quality Assessment

The quality of case-control and cohort studies was assessed using The Newcastle-Ottawa Scale (NOS), which has a maximum score of 9. Studies with a total score of 1 to 3, 4 to 6, 7 to 9 in the NOS scale were considered low, intermedia, and high quality, respectively. The quality of cross-sectional and longitudinal studies was assessed using JBI Meta-Analysis of Statistics Assessment and Review Instrument (JBI-MAStARI) (13). The JBI-MAStARI includes eight questions. The studies were classified as follows: high quality (≥5 “Yes” response); moderate quality (3–4 “Yes” response); low quality (0–2 “Yes” response) (14). Two authors conducted the quality assessment procedure independently and the disagreements were discussed by the two authors or sought help from the third author.

### Data Analysis

The meta-analysis was conducted using Review Manager 5.4 software. The heterogeneity across studies was tested by the Q statistic and *I*^2^ statistic. A *P*-value > 0.1 or an *I*^2^ statistic ≤ 50% suggested low heterogeneity across studies, and the fixed effect model was chosen. Otherwise, the random effect model was applied. For dichotomous data, the odds ratio (OR) with 95% confidence interval (CI) was used, while the weighted mean difference (WMD) with 95% CI was used for continuous data. *P*-value <0.05 were considered statistically significant.

## Results

### Study Selection

**Figure 1:**
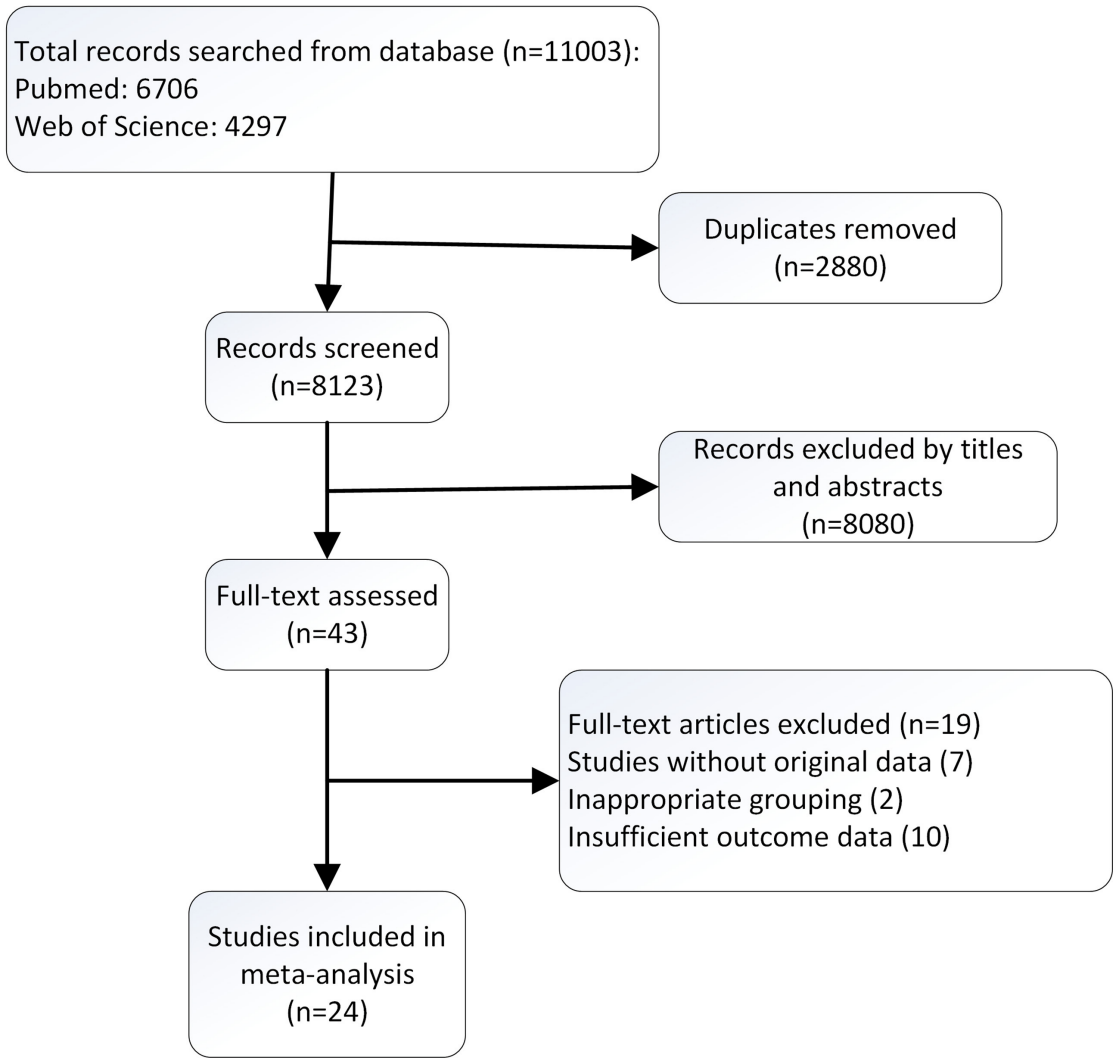
Flow diagram of reference selection process.

The flow diagram of the searching and screening process was outlined in Figure 1. A total of 24 publications with 23,845 participants were eventually included in the meta-analysis. The prevalence or incidence of sexual dysfunction was reported as the measurement of outcome variables in 21 studies (15–35).

### Study Characteristics

The sample sizes of the included studies ranged from 63 to 2,916. Detailed characteristics were listed in Table 1. The severity of LUTS in most studies was assessed by using International Prostate Symptom Score (IPSS), and the assessment of sexual dysfunction in most studies was processed based on International Index of Erectile Function (IIEF). Eighteen studies were judged to be of high quality. The details of quality assessment were shown in Supplementary Table S1.

**Table 1:** Clinical and demographic characteristics of included studies.

**Study**	**Country**	**Sample size**	**Study type**	**Age (years)**	**Criterion tools**	**Grouping criterion of experimental group**	**Grouping criterion of control group**	**Diagnosis criterion of outcomes**	**JBI-Quality score**
Dumbraveanu et al. (15)	Moldova	1,186	Cross-sectional study	18–80	IPSS/IIEF-5	IPSS score: 1–35	IPSS score: 0	—	4
Gomes et al. (16)	Brazil	2,183	Cross-sectional study	40–91	IPSS/IIEF-5	IPSS score: 1–35	IPSS score: 0	IIEF-5 score: <22	4
Kardasevic et al. (17)	Bosnia and Herzegovina	150	Cross-sectional study	40–60	IPSS/IIEF-5	IPSS score: 9–35	IPSS score: 0–8	IIEF-5 score: <22	5
Song et al. (18)	China	1,644	Cross-sectional study	64.5 ± 9.8	IPSS/IIEF-5	IPSS score: 8–35	IPSS score: 0–7	IIEF-5 score: <22	8
Demir et al. (19)	Turkey	190	Cross-sectional study	>40	IPSS/IIEF	IPSS score: 20–35	IPSS score: 8–19	IIEF score: <26	8
Ozayar et al. (20)	Turkey	453	Cross-sectional study	50–89	IPSS/IIEF	IPSS score: 20–35	IPSS score: 8–19	IIEF score: <26	7
Terai et al. (21)	Japan	2,084	Cross-sectional study	—	IPSS/IIEF	IPSS score: 8–35	IPSS score: 0–7	IIEF-5 score: <22	5
Vallancien et al. (22)	France/ Denmark/ Netherlands/ Switzerland/ United Kingdom	927	Cross-sectional study	36–92	IPSS/DAN-PSS-sex	IPSS score: 8–35	IPSS score: 0–7	—	7
Shiri et al. (23)	Finland	1,716	Cross-sectional study	mean: 58.0	DAN-PSS-1/IIEF-5	DAN-PSS-1 score:>7	DAN-PSS-1 score:0–6	IIEF-5 score: <21	6
Shiri et al. (24)	Finland	1,126	Longitudinal study	mean: 56.4	DAN-PSS-1/Two questions	DAN-PSS-1 score: >7	DAN-PSS-1 score: 0–6	Having difficulties in achieving and/or maintaining an erection	6
Mak et al. (25)	Belgium	799	Cross-sectional study	40–69	IPSS/IIEF-question 15	IPSS score: 8–35	IPSS score: 0–7	Having difficulties in achieving and/or maintaining an erection	7
Nicolosi et al. (26)	Brazil/ Italy/ Japan/ Malaysia	2,290	Cross-sectional study	40–70	IPSS/One question	IPSS score: 8–35	IPSS score: 0–7	Sometimes/never able to get and keep an erection adequate for satisfactory sexual intercourse	3
Adegun et al. (27)	Nigeria	303	Cross-sectional study	Case: 66.03 ± 9.64 Control: 65.78 ± 8.61	Clinical diagnosis/IIEF	Patients diagnosed with LUTS	Patients diagnosed without LUTS	IIEF score: <60	5
Wang et al. (28)	China	400	Cross-sectional study	50–80	IPSS/IIEF-5	IPSS score: 8–35	IPSS score: 0–7	IIEF-5 score: <22	7
Li et al. (29)	Singapore/ Philippines/ Hongkong/ Malaysia/ Thailand	1,155	Cross-sectional study	50–80	IPSS/DAN-PSS-sex	IPSS score: 8–35	IPSS score: 0–7	__	5
Naya et al. (30)	Japan	250	Cross-sectional study	Case: mean 33.2 Control: mean 32.5	IPSS/IIEF-5	IPSS score: 8–35	IPSS score: 0–7	IIEF-5 score: <17	3
Zhang et al. (31)	China	1,406	Cross-sectional study	18–60	NIH-CPSI/IIEF-5	NIH-CPSI score: 15–43	NIH-CPSI score: 5–14	IIEF-5 score: <22	5
Dogan et al. (32)	Turkey	78	Cross-sectional study	45–84	IPPS/IIEF-5	IPSS score: 8–35	IPSS score: 0–7	IIEF-5 score: 6–25	6
Mo et al. (33)	China	640	Cross-sectional study	Case: 28.95 ± 4.98 Control: 27.6 ± 3.85	NIH-CPSI/IIEF-5	NIH-CPSI score: ≥5	NIH-CPSI score: <5	IIEF-5 score: <22	5
Sonmez et al. (34)	Turkey	63	Cross-sectional study	Case: 22–48 Control: 24–48	(NIH-CPSI + clinical diagnosis)/IIEF	NIH-CPSI score: ≥14	patients without any sign of urological infection and any symptom of pain or disturbance	—	6
Rhoden et al. (35)	Brazil	192	Cross-sectional study	40–81	IPSS/IIEF	IPSS score: 8–35	IPSS score: 0–7	IIEF score: <25	8
Fwu et al. (36)	USA	2,916	Cross-sectional study	62.6 ± 7.3	AUA-SI/BMSFI	AUA-SI score: ≥20	AUA-SI score: 8–19	—	4
Gao et al. (37)	China	1,280	Cross-sectional study	34.5 ± 9.20	NIH-CPSI/IIEF-5	NIH-CPSI score: ≥10	NIH-CPSI score: 0-9	—	7
Macnab et al. (38)	African	414	Cross-sectional study	64.9 ±12.3	IPSS/Epstein Inventory	IPSS score: 8–35	IPSS score: 0–7	—	4

*IPSS, international prostate symptom score; IIEF-5, 5-item International Index of Erectile Function; IIEF, International Index of Erectile Function; DAN-PSS, Danish Prostatic Symptom Score; NIH-CPSI, National Institutes of Health—Chronic Prostatitis Symptom Index; AUA-SI, American Urological Association—Symptom Index; BMSFI, Brief Male Sexual Function Inventory*.

### Evidence Synthesis

#### Overall Assessment of Sexual Dysfunction

**Figure 2:**
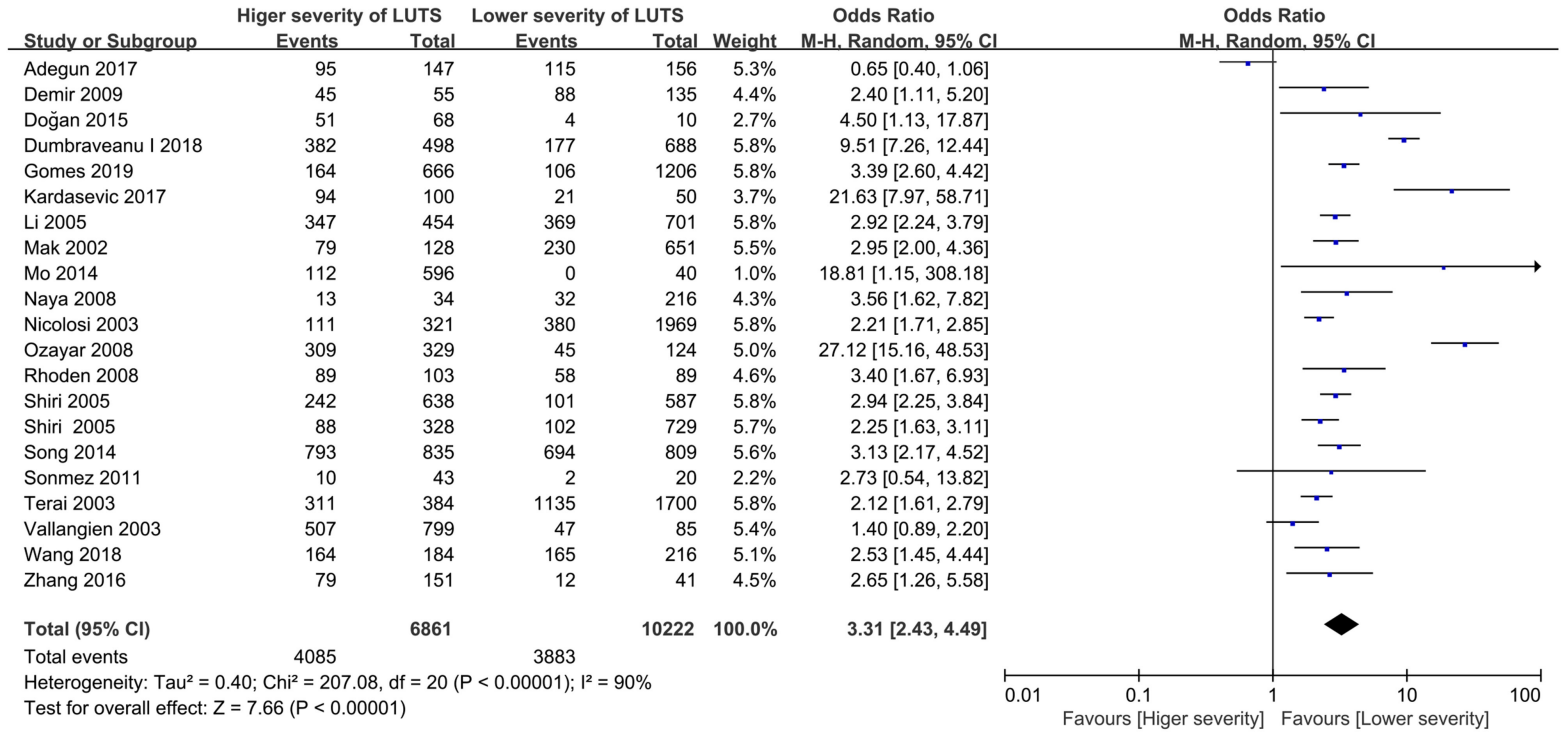
Forest plot showing meta-analysis results of the association between LUTS severity and sexual dysfunction prevalence using dichotomous data. LUTS, lower urinary tract symptoms; CI, confidence interval.

Twenty-one studies (15–35) reported the number of sexual dysfunction cases in participants with different severities of LUTS. The group with high scores of scales for LUTS named the group with higher severity of LUTS and the group with lower scores of scales for LUTS named the group with lower severity of LUTS. The result indicated that participants with higher severity of LUTS also had a higher prevalence of sexual dysfunction compared with participants with lower severity (OR = 3.31, 95% CI: 2.43 to 4.49, *p* < 0.001, Figure 2). Heterogeneity across studies was high (*I*^2^ = 90%, *P* < 0.001).

**Figure 3:**
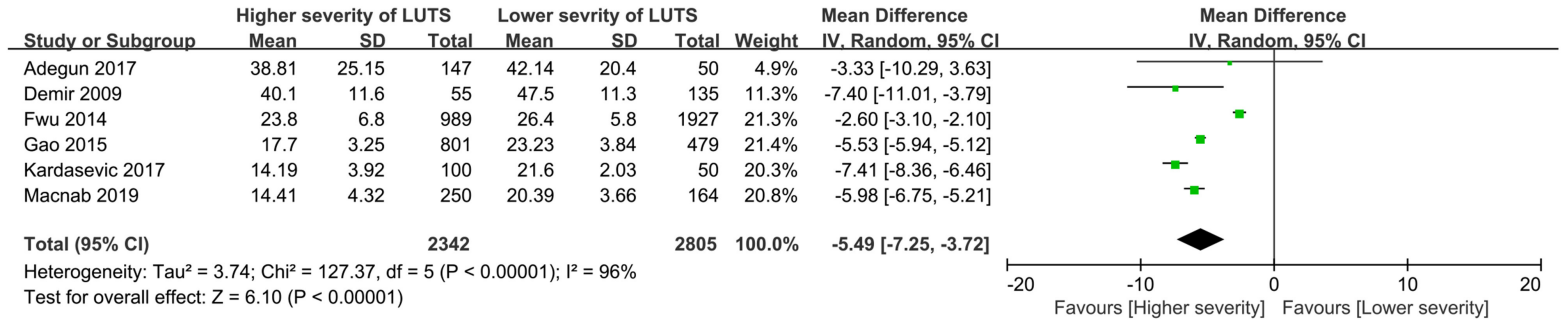
Forest plot showing meta-analysis results of the association between LUTS severity and sexual dysfunction scoring using continuous data. LUTS, lower urinary tract symptoms; CI, confidence interval; SD, standard deviation.

Six studies (17, 19, 27, 36–38) reported scores of assessment tools for sexual dysfunction as a measurement of sexual dysfunction in participants with different severities of LUTS. The result suggested that men with higher severity of LUTS had lower scores of assessment tools for sexual dysfunction compared with those with lower severity (WMD = −5.49, 95%CI: −7.25 to −3.27, *P* < 0.001, Figure 3). Heterogeneity across studies was significant (*I*^2^ = 96%, *P* < 0.001).

#### Assessment of Sexual Function Domains

**Figure 4:**
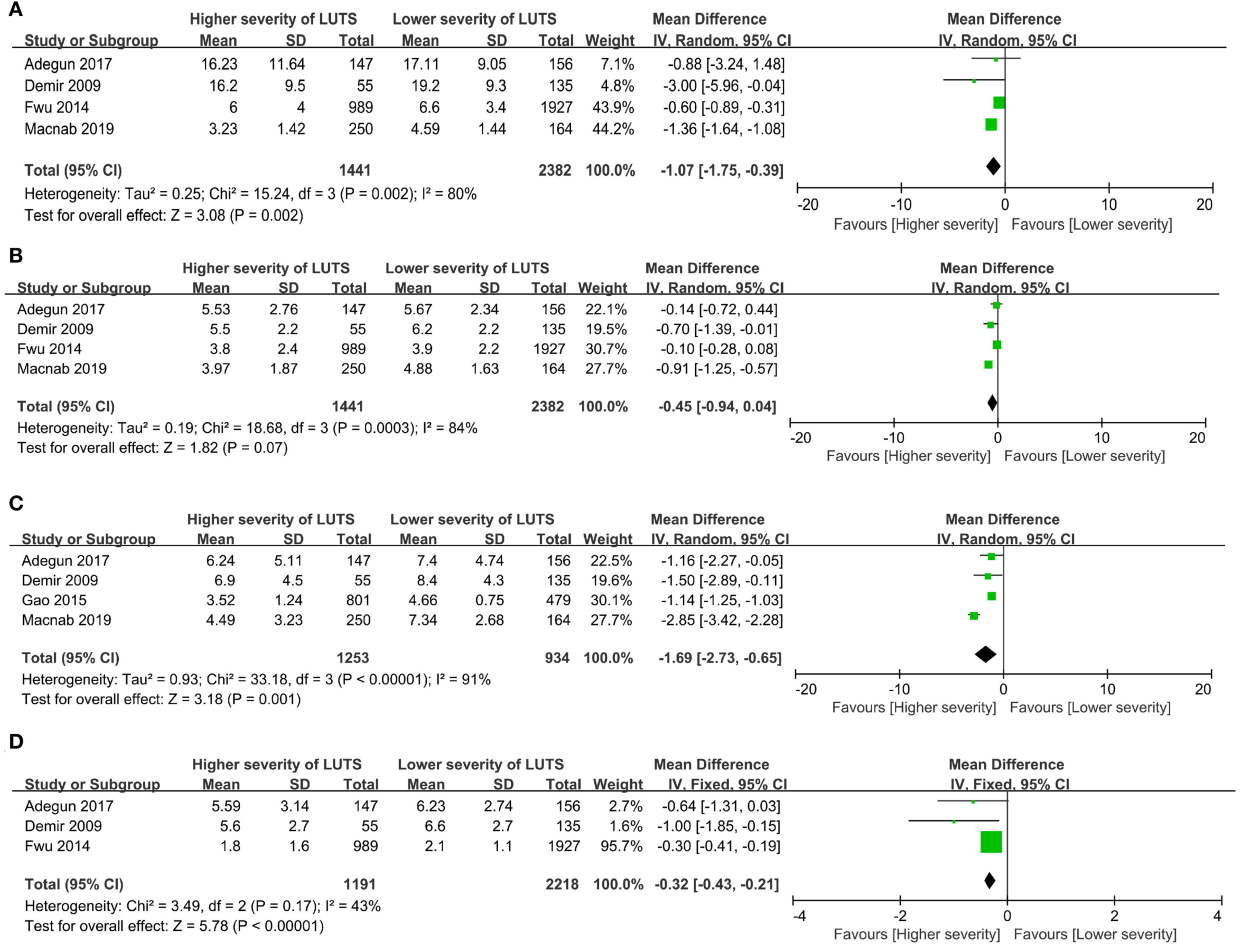
Forest plots showing meta-analysis results of the association between LUTS severity and the sexual dysfunction domains using Sexual dysfunction assessment tools. **(A)** Erectile function, **(B)** Sexual desire, **(C)** Intercourse satisfaction and **(D)** Overall satisfaction. LUTS, lower urinary tract symptoms; CI, confidence interval; SD, standard deviation.

Four studies reported the erectile function domain in their results (19, 27, 36, 38). The result indicated that the participants with higher severity of LUTS had worse erectile function than participants with lower severity (WMD = −1.07, 95%CI: −1.75 to −0.39, *P* = 0.002, Figure 4A).

Four studies evaluated the sexual desire of patients with different severity of LUTS (19, 27, 36, 38). The result demonstrated that the difference of sexual desire among participants with different severities of LUTS was not statistically significant (WMD = −0.45, 95%CI: −0.94 to 0.04, *P* = 0.07, Figure 4B).

Four studies evaluated the intercourse satisfaction domain (19, 27, 37, 38). The result indicated that participants with higher severity of LUTS had lower intercourse satisfaction compared with participants with lower severity (WMD = −1.69, 95%CI: −2.73 to −0.65, *P* = 0.001, Figure 4C).

Three studies evaluated the overall satisfaction domain (19, 27, 36). The result indicated that the participants with higher severity of LUTS had lower overall satisfaction compared with participants with lower severity (WMD = −0.32, 95%CI: −0.43 to −0.21, *P* < 0.001, Figure 4D).

#### Subgroup Analysis

Subgroup-analysis was performed according to age, assessment tools for LUTS and assessment tools for sexual function.

In subgroup-analysis, the results indicated that the prevalence of sexual dysfunction in participants with higher severity of LUTS was higher compared with participants with lower severity of LUTS in both participants with age ≤40 years (OR= 3.54, 95%CI: 1.58 to 7.95, *P* = 0.002) and participants with age >40 years (OR = 3.94, 95%CI: 2.80 to 5.53, *P* < 0.001). Inter-study heterogeneity was high and significant in both groups (*I*^2^ = 61%, and *I*^2^ = 89%). The pooled analysis forest plot is shown in Supplementary Figure 1.

To assess the severity of LUTS, IPSS was used in 15 studies (15–22, 25, 26, 28–30, 32, 35), DAN-PSS-1 was used in two studies (23, 24) and NIH-CPSI was used in three studies (31, 33, 34). In one study (27), LUTS was assessed by clinical diagnosis. The results indicated that the prevalence of sexual dysfunction in participants with higher severity of LUTS was higher compared with participants with lower severity of LUTS in the groups assessing LUTS with IPSS, DAN-PSS-1 and NIH-CPSI (IPSS group: OR = 3.83, 95%CI: 2.68 to 5.48, *P* < 0.001, *I*^2^ = 91%; DAN-PSS-1 group: OR = 2.61, 95%CI: 2.02 to 3.39, *P* < 0.001, *I*^2^ = 36%; NIH-CPSI group: OR = 3.07, 95%CI: 1.43 to 6.58, *P* = 0.004, *I*^2^ = 9%). Analysis of the study that used clinical diagnosis to assess LUTS revealed that there was no association between exposure of LUTS and risk of sexual dysfunction (OR = 0.65, 95%CI: 0.40 to 1.06, *P* = 0.09). The pooled analysis forest plot is shown in Supplementary Figure 2.

In those studies which reported scores of assessment tools for sexual dysfunction, 4 assessment tools had been used to measure sexual dysfunction. Five-item International Index of Erectile Function (IIEF-5), IIEF, Brief Male Sexual Function Inventory (BMSFI) and Epstein Inventory were used to assess sexual dysfunction in two studies (17, 37), two studies (19, 27), one study (36) and one study (38), respectively. The results showed that participants with higher severity of LUTS had lower scores of the assessment tools than participants with lower severity of LUTS in all groups (IIEF-5 group: WMD = −6.42, 95%CI: −8.26 to −4.58, *P* < 0.001, *I*^2^ = 92%; IIEF group: WMD = −5.80, 95%CI: −9.70 to −1.91, *P* = 0.003, *I*^2^ = 37%; BMSFI group: WMD = −2.60, 95%CI: −3.10 to −2.10, *P* < 0.001; Epstein inventory group: WMD = −5.98, 95%CI: −6.75 to −5.21, *P* < 0.001). The pooled analysis forest plot is shown in Supplementary Figure 3.

## Discussion

In our meta-analysis, the result revealed that participants with higher severity of LUTS had a higher prevalence of sexual dysfunction compared with those participants with lower severity of LUTS. Specifically, the result indicated that participants with higher severity of LUTS had worse erectile function, intercourse satisfaction, and overall satisfaction compared with those participants with lower severity of LUTS. Therefore, LUTS may impact sexual activity including erectile function and sexual satisfaction.

According to previous studies, age may be a significant confounding factor of sexual dysfunction (39). However, the difference in the prevalence of sexual dysfunction between subgroups with different ages was not statistically significant. The reason may be that the number of studies and participants included in age ≤40 years subgroup was limited. According to the results of subgroup analysis, the assessment tools for LUTS and sexual dysfunction can also contribute to the heterogeneity. In addition, the high heterogeneity may also result from variations in study country, population selected, comorbidities, medical history, and history of surgery. Different investigators and different survey methods among studies will also result in heterogeneity. The cross-tabulation analyses in studies represented the original source of effect size, whereas the effect sizes determined by ORs and 95%CIs in some studies were adjusted according to confounding factors such as age, comorbidities, and other factors. Therefore, the results determined by ORs were more reliable and credible (40).

A prospective study performed by Alison et.al has shown that the risk of ED increased with LUTS severity (41). Due to the lack of original data, this study wasn't included in our review. Moreover, population-based studies also have shown that ED increases the risk of LUTS (42, 43). Combined with our result, the causality between LUTS and ED may be bidirectional. Sexual dysfunction and LUTS may share common pathophysiological mechanisms. The mechanism underlying the association between LUTS and sexual dysfunction remains to be established. However, some hypothesizes have been proposed according to the results of published studies. The most acceptable mechanism is the lack of nitric oxide (NO). The decrease of NO/cyclic guanosine monophosphate (cGMP) will cause a reduction in NO synthase because of endothelial dysfunction and it will result in erectile dysfunction due to unnormal regulation of penile corporal smooth relaxation. Reduction of smooth muscle relaxation of bladder neck, prostate, and urethra may also cause LUTS (44). RhoA/rho-kinase-calcium-sensitizing pathway may also play a role in the occurrence of LUTS and sexual dysfunction. Activation of the RhoA/rho-kinase-calcium-sensitizing pathway can affect smooth muscle relaxation, which can cause LUTS and sexual dysfunction (45). Autonomic hyperactivity may be also associated with LUTS and sexual dysfunction. Increased autonomic activity will up-regulate the number of α_1_-adrenoceptors and the secretion of noradrenaline, which can mediate adrenergic contraction of smooth muscles in the bladder neck, prostate, urethra, and the corpus cavernosum (12). The vascular disease such as pelvic atherosclerosis will result in chronic ischemia and impair neurogenic relaxation in the prostate, corpus cavernosum and bladder neck. The association between sexual dysfunction and LUTS has biological plausibility, and the mechanisms are not mutually exclusive and independent. For example, atherosclerosis-induced pelvic ischemia will increase autonomic activity, reduce NO production, and upregulate Rho kinase, leading to the dysfunction of penile smooth muscle, and bladder ischemia, which contributes to bladder outlet obstruction or pelvic vascular disease. In the end, sexual dysfunction and LUTS would happen (46). In addition, psychological factors could also contribute to both LUTS and sexual dysfunction. Autonomic activity could be the main reason behind psychogenic ED and contribute to the development of LUTS. Stress, depression and anxiety will lead to LUTS and sexual dysfunction by the mechanism of increased autonomic activity (47). According to our result, LUTS affected erectile function, sexual satisfaction and overall satisfaction, and had no significant effect on sexual desire.

Several medications including phosphodiesterase inhibitors, alpha blockers, 5 alpha inhibitors and testosterone are applied to manage LUTS and sexual dysfunction patients. Phosphodiesterase type 5 (PDE5) is expressed in the whole of the lower urinary tract, including the urethra, prostate, and bladder (48). PDE5 is localized in endothelial and smooth muscle cells, suggesting the action of PDE5- inhibitors (PDE5i) on smooth muscle contraction and blood flow. According to some studies, PDE5i had significant safety and efficacy on LUTS and sexual dysfunction (49, 50). Alpha blockers and 5 alpha reductase inhibitors are used for the management of LUTS. They are both effective as a single treatment or in combination, but that treatment might bring side effects on ejaculatory and sexual function (51). The possible role of testosterone has been evaluated. Long-term testosterone therapy in hypogonadal men resulted in significant improvements in urinary and sexual function and quality of life (52). Phytotherapy has also been investigated. A formulation containing *Serenoa repens, Crocus sativus*, and *Pinus massoniana* extracts has been used to therapy patients with LUTS and ED and the results indicated LUTS and ED improved after 90 days of treatment (53).

The most important strength of this meta-analysis is that the result may be a reliable evidence to indicate the association between LUTS and sexual dysfunction. This meta-analysis is the first to investigate the association between LUTS and sexual dysfunction. Therefore, the result is useful for clinicians, policymakers and patients. For patients with sexual dysfunction, the probability of concurrent LUTS should be considered and assessed. The treatment of LUTS may help the treatment of sexual dysfunction.

There are also some limitations. First, we only searched the literature in two databases and some relevant papers in other databases, such as Embase, may be missed. Although we searched Embase after original systematic review and found no additional suitable article. Second, although subgroup analyses were conducted, the high heterogeneity due to the differences of assessment tools among studies made it difficult to generalize the conclusion. Furthermore, the study types of the included studies only include cross-sectional study and longitudinal study, so the result of the meta-analysis cannot determine the causality between LUTS and sexual dysfunction. Further cohort and case-control studies should be performed to investigate the relationship between LUTS and sexual dysfunction.

## Conclusion

Males with higher severity of LUTS had worse sexual function compared with those with lower severity of LUTS. The result also gives a remind to clinicians, policymakers, and patients that exposure to LUTS may also have a high probability of sexual dysfunction, and they should pay attention to the accidence of sexual dysfunction.

## Data Availability Statement

The original contributions presented in the study are included in the article/Supplementary Material, further inquiries can be directed to the corresponding author/s.

## Author Contributions

GS and MW contributed to data acquisition. XL and YZ came up with the idea of doing this research and are responsible for the whole work. GS, MW, BC, GL, HL, RL, ZL, CW, TW, SW, and JL participated in data analysis, writing, and revision of the article. All authors read and approved the final version of the manuscript.

## Conflict of Interest

The authors declare that the research was conducted in the absence of any commercial or financial relationships that could be construed as a potential conflict of interest. The reviewer XY declared a shared affiliation, with no collaboration, with the authors to the handling editor at the time of the review.

## Abbreviations

LUTS: lower urinary tract symptoms
ED: erectile dysfunction
OR: odds ratio
MD: mean difference
CI: confidence interval
IPSS: international prostate symptom score
IIEF: international index of erectile function.
