# Supplementary material for: Lower Urinary Tract Symptoms and Sexual Dysfunction in Male: A Systematic Review and Meta-Analysis
Source: Front Med (Lausanne). 2021 May 28;8:653510. doi: 10.3389/fmed.2021.653510 (PMC8193225; doi:10.3389/fmed.2021.653510)
Supplement: Supplementary file 1 [file Data_Sheet_1.DOCX]

**supplementary appendix**

Questionnaires for assessments of LUTS and sexual function

International Prostate Symptom Score (IPSS)…………………………………….2

Danish Prostatic Symptom Score (DAN-PSS-1) ………………………………....4

NIH CHRONIC PROSTATITIS SYMPTOM INDEX (NIH-CPSI) …………..…6

Individual items of International Index of Erectile Function (IIEF) ……………..8

A brief sexual function inventory (BSFI) …………………………………….....10

Epstein Inventory …………………………………………………………….…12

Danish Prostatic Symptom Score (DAN-PSS-sex) …………………………..…13

Table S1 Risk of bias within studies using the Joanna Briggs Institute criteria’s..………………14

Fig S1…………………………………………………………………………………………15

Fig S2…………………………………………………………………………………………16

Fig S3…………………………………………………………………………………………17

**International Prostate Symptom Score (IPSS)**

| **In the past month:** | Not at All | Less than 1 in 5 Times | Less than Half the Time | About Half the Time | More than Half the Time | Almost Always | Your score |
| --- | --- | --- | --- | --- | --- | --- | --- |
| **1.Incomplete Emptying** How often have you had the sensation of not emptying your bladder? | 0 | 1 | 2 | 3 | 4 | 5 |  |
| **2.Frequency** How often have you had to urinate less than every two hours? | 0 | 1 | 2 | 3 | 4 | 5 |  |
| **3.Intermittency** How often have you found you stopped and started again several times when you urinated? | 0 | 1 | 2 | 3 | 4 | 5 |  |
| **4.Urgency** How often have you found it difficult to postpone urination? | 0 | 1 | 2 | 3 | 4 | 5 |  |
| **5.Weak Stream** How often have you had a weak urinary stream? | 0 | 1 | 2 | 3 | 4 | 5 |  |
| **6.Straining** How often have you had to strain to start urination? | 0 | 1 | 2 | 3 | 4 | 5 |  |
|  | None | 1 Time | 2 Time | 3 Time | 4 Time | 5 Time |  |
| **7. Nocturia** How many times did you typically get up at night to urinate? | 0 | 1 | 2 | 3 | 4 | 5 |  |
| **Total I-PSS Score** |  |  |  |  |  |  |  |

**Score:** 1-7: **Mild** 8-19: **Moderate**  20-35: **Severe**

| **Quality of Life Due to Urinary Symptoms** | Delighted | Pleased | Mostly Satisfied | Mixed | Mostly Dissatisfied | Unhappy | Terrible |
| --- | --- | --- | --- | --- | --- | --- | --- |
| If you were to spend the rest of your life with your urinary condition just the way it is now, how would you feel about that? | 0 | 1 | 2 | 3 | 4 | 5 | 6 |

**Danish Prostatic Symptom Score (****DAN-PSS-1)**

The DAN-PSS-1 questionnaire. Each question allows the patient to choose one of four answers. For each question, the patient scores 0-3 for severity of symptoms (A) and 0-3 for the degree of bother (B).

**1A** *Hesitancy*: Do you have to wait for urination to start?

Answers: 0-No; 1-Rarely; 2-Daily; 3-Every time

**1B** If you have to wait to start urination, is this a problem for you?

Answers: 0-No problem; 1-Small problem; 2-Moderate problem; 3-Major problem

**2 A** *Weak stream*: Do you consider your urinary stream as:

Answers: 0-Normal; 1-Weak; 2-Very weak; 3-Dribbling

**2B** If your stream is weak or dribbling, is this a problem for you?

Answers: 0-No problem; 1-Small problem; 2-Moderate problem; 3-Major problem

**3A** *Incomplete emptying*: Do you feel you empty your bladder completely?

Answers: 0-Always; 1-Occasionally; 2-Rarely; 3-Never

**3B** If you feel that you do not empty your bladder completely, is this a problem for you?

Answers: 0-No problem; 1-Small problem; 2-Moderate problem; 3-Major problem

**4A** *Straining*: Do you have to strain to start and/or maintain urination?

Answers: 0-No; 1-Rarely; 2-Daily; 3-Always

**4B** If you have to strain, is this a problem for you?

Answers: 0-No problem; 1-Small problem; 2-Moderate problem; 3-Major problem

**5A** *Daytime frequency*: What is the longest interval between each urination, from when you wake up until you go to bed?

Answers: 0-More than three hours: 1-Two to three hours; 2-One to two hours; 3-Less than one hour

**5B** Do you consider your frequency of urination a problem?

Answers: 0-No problem; 1-Small problem; 2-Moderate problem; 3-Major problem

**6A** *Nocturia*: How many times do you have to urinate during the night?

Answers: 0-None; 1-One to two times; 2-Three to four times; 3-Five times or more

**6B** If you have to urinate during the night, is this a problem for you?

Answers: 0-No problem; 1-Small problem; 2-Moderate problem; 3-Major problem

**7A** *Urge*: Do you experience an imperative (strong) urge to urinate?

Answers: 0-Never; 1-Rarely; 2-Daily; 3-Always

**7B** If you have an imperative (strong) urge to urinate, is this a problem for you?

Answers: 0-No problem; 1-Small problem; 2-Moderate problem; 3-Major problem

**8A** *Urge incontinence*: Is the urge to urinate so strong that urine starts to flow before you reach the toilet?

Answers: 0-Never; 1-Rarely; 2-Daily; 3-Every time

**8B** If the urge to urinate is so strong that urine starts to flow before you reach the toilet, is this a problem for you?

Answers: 0-No problem; 1-Small problem; 2-Moderate problem; 3-Major problem

**9A** *Dysuria*: Do you feel pain or have a burning feeling when you urinate?

Answers: 0-Never; 1-Rarely; 2-Daily; 3-Always

**9B** If it hurts or burns when you urinate, is this a problem for you?

Answers: 0-No problem; 1-Small problem; 2-Moderate problem; 3-Major problem

**10A** *Post-micturition dribbling*: Do you experience dribbling after voiding, when you feel you have finished urination?

Answers: 0-Never; 1-In the toilet; 2-Small amounts in the trousers; 3-Large amounts in the trousers

**10B** If you experience dribbling after voiding, is this a problem for you?

Answers: 0-No problem; 1-Small problem; 2-Moderate problem; 3-Major problem

**11A** *Stress incontinence*: Do you experience leakage of urine when physically active (e.g., lifting, sneezing, coughing)?

Answers: 0-Never; 1-Rarely; 2-Often; 3-Always

**11B** If you experience urinary leakage when physically active, is this a problem for you?

Answers: 0-No problem; 1-Small problem; 2-Moderate problem; 3-Major problem

**12A** *Overflow/Seeping incontinence*: Do you experience leakage of urine without urge or physical activity?

Answers: 0-Never; 1-Rarely; 2-Often; 3-Always

**12B** If you experience urinary leakage without urge or physical activity, do you consider this a problem?

Answers: 0-No problem; 1-Small problem; 2-Moderate problem; 3-Major problem

**NIH CHRONIC PROSTATITIS SYMPTOM INDEX (NIH-CPSI)**

Pain or Discomfort

1. In the last week, have you experienced any pain or discomfort in the following areas?

Yes No

○1 ○0

○1 ○0

○1 ○0

○1 ○0

a. Area between rectum and testicles (perineum)

b. Testicles

c. Tip of the penis (not related to urination)

d. Below your waist, in your pubic or bladder area

1. In the last week, have you experienced:

Yes No

○1 ○0

○1 ○0

a. Pain or burning during urination?

b. Pain or discomfort during or after sexual climax (ejaculation)?

1. How often have you had pain or discomfort in any of these areas over the last week?

○0 Never

○1 Rarely

○2 Sometimes

○3 Often

○4 Usually

○5 Always

1. Which number best describes your AVERAGE pain or discomfort on the days that you had it, over the last week?

○0 ○1 ○2 ○3 ○4 ○5 ○6 ○7 ○8 ○9 ○10

NO PAIN PAIN AS BAD AS YOU CAN IMAGINE

Urination

1. How often have you had a sensation of not emptying your bladder completely after you finished urinating, over the last week?

○0 Not at all

○1 Less than 1 time in 5

○2 Less than half the time

○3 About half the time

○4 More than half the time

○5 Almost always

1. How often have you had to urinate again less than two hours after you finished urinating, over the last week?

○0 Not at all

○1 Less than 1 time in 5

○2 Less than half the time

○3 About half the time

○4 More than half the time

○5 Almost always

Impact of Symptoms

1. How much have your symptoms kept you from doing the kinds of things you would usually do, over the last week?

○0 None

○1 Only a little

○2 Some

○3 A lot

1. How much did you think about your symptoms, over the last week?

○0 None

○1 Only a little

○2 Some

○3 A lot

Quality of Life

1. If you were to spend the rest of your life with your symptoms just the way they have been during the last week, how would you feel about that?

○0 Delighted

○1 Pleased

○2 Mostly satisfied

○3 Mixed (about equally satisfied and dissatisfied)

○4 Mostly dissatisfied

○5 Unhappy

○6 Terrible

Scoring the NIH-Chronic Prostatitis Symptom Index Domains

Pain: Total of items la, lb, lc, Id, 2a, 2b, 3**,** and 4 =

Urinary Symptoms: Total of items ***5*** and 6 =

Quality of Life Impact: Total of items 7, 8 and 9 =

**Individual items of International Index of Erectile Function (IIEF)** Questionnaire and response options (US version)

| **Question*** | **Response Options** |
| --- | --- |
| Q1 : How often were you able to get an erection during sexual activity? Q2: When you had erections with sexual stimulation, how often were your erections hard enough for penetration? | 0 = No sexual activity 1 = Almost never/never 2 = A few times (much less than half the time) 3 = Sometimes (about half the time) 4 = Most times (much more than half the time) 5 = Almost always/always |
| Q3: When you attempted sexual intercourse, how often were you able to penetrate (enter) your partner? Q4: During sexual intercourse, how often were you able to maintain your erection after you had penetrated (entered) your partner? | 0 = Did not attempt intercourse 1= Almost never/never 2= A few times (much less than half the time) 3 = Sometimes (about half the time) 4 = Most times (much more than half the time) 5 = Almost always/always |
| Q5: During sexual intercourse, how difficult was it to maintain your erection to completion of intercourse? | 0 = Did not attempt intercourse 1 = Extremely difficult 2 = Very difficult 3 = Difficult 4 = Slightly difficult 5 = Not difficult |
| Q6: How many times have you attempted sexual intercourse? | 0 = No attempts 1 = One to two attempts 2 = Three to four attempts 3 = Five to six attempts 4 = Seven to ten attempts 5 = Eleven+ attempts |
| Q7: When you attempted sexual intercourse, how often was it satisfactory for you? | 0 = Did not attempt intercourse 1 = Almost never/never 2 = A few times (much less than half the time) 3 = Sometimes (about half the time) 4 = Most times (much more than half the time) 5 = Almost always/always |
| Q8: How much have you enjoyed sexual intercourse? | 0 = No intercourse 1 = No enjoyment 2 = Not very enjoyable 3 = Fairly enjoyable 4 = Highly enjoyable 5 = Very highly enjoyable |
| Q9: When you had sexual stimulation or intercourse, how often did you ejaculate? Q10. When you had sexual stimulation or intercourse, how often did you have tile feeling of orgasm or climax? | 0 = No sexual stimulation/intercourse 1 = Almost never/never 2 = A few times (much less than half the time) 3 = Sometimes (about half the time) 4 = Most times (much more than half the time) 5 = Almost always/always |
| Q11: How often have you felt sexual desire? | 1 = Almost never/never 2 = A few times (much less than half the time) 3 = Sometimes (about half the time) 4 = Most times (much more than half the time) 5 = Almost always/always |
| Q12: How would you rate your level of sexual desire? | 1 = Very low/none at all 2 = Low 3 = Moderate 4 = High 5 = Very high |
| Q13: How satisfied have you been with your overall sex life? Q14: How satisfied have you been with your sexual relationship with your partner? | 1 = Very dissatisfied 2 = Moderately dissatisfied 3 = About equally satisfied and dissatisfied 4 = Moderately satisfied 5 = Very satisfied |
| Q15: How do you rate your confidence that you could get and keep an erection? | 1 = Very low 2 = Low 3 = Moderate 4 = High 5 = Very high |
| * All questions are preceded by the phrase "Over the past 4 weeks." | |
|  |  |

**A brief sexual function inventory (BSFI)**

| **SEXUAL DRIVE** | | | | | |
| --- | --- | --- | --- | --- | --- |
| Let’s define sexual drive as a feeling that may include wanting to have a sexual experience (masturbation or intercourse), thinking about having sex, or feeling frustrated due to lack of sex. 1. During the past 30 days, on how many days have you felt sexual drive? | No days | only a few days | Some days | Most days | Almost every day |
|  | 0 | 1 | 2 | 3 | 4 |
| 2. During the past 30 days. how would you rate your level of sexual drive? | None at all | Low | Medium | Medium High | High |
|  | 0 | 1 | 2 | 3 | 4 |
| **ERECTIONS** | | | | | |
| 3. Over the past 30 days, how often have you had partial or full sexual erections. when you were sexually stimulated in any way? | Not at all | A few times | Fairly often | Usually | Always |
|  | 0 | 1 | 2 | 3 | 4 |
| 4. Over the past 30 days. when you had erections, how often were they firm enough to have sexual intercourse? | 0 | 1 | 2 | 3 | 4 |
| 5. How much difficulty did you have getting an erection during the past 30 days? | Did not get erections at all | A lot of difficulty | Some difficulty | Little difficulty | No difficulty |
|  | 0 | 1 | 2 | 3 | 4 |
| **EJACULATlON** | | | | | |
| 6. In the paat 30 days, how much difficulty have you had ejaculating when you have been sexually stimulated? | Have had no sexual stimulation in past month | A lot of difficulty | Some difficulty | Little difficulty | No difficulty |
|  | 0 | 1 | 2 | 3 | 4 |
| 7. ln the past 30 days, how much did you consider the amount of semen you ejaculate to be a problem for you? | Did not climax | Big problem | Medium problem | Small problem | No problem |
|  | 0 | 1 | 2 | 3 | 4 |
| **PROBLEM ASSESSMENT** | | | | | |
| 8. In the past 30 days. to what extent have you considered a lack of sex drive to be a problem? | Big problem | Medium problem | Small problem | Very small problem | No problem |
|  | 0 | 1 | 2 | 3 | 4 |
| 9. In the past 30 days, to what extent have you considered your ability to get and keep erection to be a problem? | 0 | 1 | 2 | 3 | 4 |
| 10. In the past 30 days, to what extent have you considered your ejaculation to be a problem? | 0 | 1 | 2 | 3 | 4 |
| **OVERALL SATISFACTION** | | | | | |
| 11. Overall, during the past 30 days, how satisfied have you been with your sex life? | Very dissatisfied | Mostly dissatisfied | Neutral or mixed (about equally satisfied and dissatisfied) | Mostly satisfied | Very satisfied |
|  | 0 | 1 | 2 | 3 | 4 |

**Epstein Inventory**

1. How satisfied are you with your level of sexual activity or lack of sexual activity?

○0 ○1 ○2 ○3 ○4 ○5 ○6 ○7 ○8 ○9 ○10

Extremely Dissatisfied very satisfied

1. During the PAST MONTH, how frequently have you felt sexual drive? This feeling may include wanting to have sex (masturbation or intercourse), planning to have sex, feeling frustrated due to lack of sex, etc.

○1 Not at all

○2 Once this month

○3 2-3 times this week

○4 Once a week

1. During the PAST MONTH, how often have you been able to have erections when you were sexually stimulated?

○1 All of the time

○2 Most of the time

○3 A good bit of the time

○4 Some of the time

○5 A little of the time

○6 None of the time

1. Over the PAST YEAR, would you say your sexual performance has gotten worse, stayed the same or better?

○1 Much worse

○2 Somewhat worse

○3 A little worse

○4 About the same

○5 A little better

○6 Somewhat better

○7 Much better

**Danish Prostatic Symptom Score (DAN-PSS-sex)**

Q1. Can you get an erection? CIRCLE ONE ANSWER ONLY.

○1 Yes, with a normal stiffness

○2 Yes, with a slight reduction in stiffness

○3 Yes, with a very reduced amount of semen

○4 No ejaculations

Q2. If you have difficulty getting an erection, how bothersome is this for you? CIRCLE ONE ANSWER ONLY.

○1 Not at all

○2 A little bit

○3 Moderately

○4 Very much

Q3. Do you have ejaculations? CIRCLE ONE ANSWER ONLY.

○1 Yes, with a normal amount of semen

○2 Yes, with a slightly reduced amount of semen

○3 Yes, with a very reduced amount of semen

○4 No ejaculations

Q4. If you ejaculate with a reduced amount of semen or if you do not ejaculate at all, how bothersome is this for you? CIRCLE ONE ANSWER ONLY.

○1 Not at all

○2 A little bit

○3 Moderately

○4 Very much

Q5. If you have ejaculations, do you experience any pain / discomfort when ejaculating? CIRCLE ONE ANSWER ONLY.

○1 No pain/discomfort

○2 Yes, slight pain/discomfort

○3 Yes, moderate pain/discomfort

○4 Strong pain/discomfort

Q6. If you experience pain / discomfort when ejaculating, how bothersome is this for you? CIRCLE ONE ANSWER ONLY.

○1 Not at all

○2 A little bit

○3 Moderately

○4 Very much

Table S1: Risk of bias within studies using the Joanna Briggs Institute criteria’s

| Included Studies | JBI quality assessment criteria’s | | | | | | | | Total score |
| --- | --- | --- | --- | --- | --- | --- | --- | --- | --- |
|  | ① | ② | ③ | ④ | ⑤ | ⑥ | ⑦ | ⑧ |  |
| Dumbraveanu I 2018 | N | N | U | Y | Y | Y | U | Y | 4 |
| Gomes 2019 | N | N | U | Y | Y | Y | U | Y | 4 |
| Kardasevic 2017 | Y | N | Y | Y | N | N | Y | Y | 5 |
| Song 2014 | Y | Y | Y | Y | Y | Y | Y | Y | 8 |
| Demir 2009 | Y | Y | Y | Y | Y | Y | Y | Y | 8 |
| Ozayar 2008 | Y | N | Y | Y | Y | Y | Y | Y | 7 |
| Terai 2003 | N | Y | U | Y | Y | Y | U | Y | 5 |
| Vallangien 2003 | Y | U | Y | Y | Y | Y | Y | Y | 7 |
| Shiri 2005 | Y | Y | U | Y | Y | Y | U | Y | 6 |
| Shiri 2005 | Y | Y | U | Y | Y | Y | U | Y | 6 |
| Mak 2002 | N | Y | Y | Y | Y | Y | Y | Y | 7 |
| Nicolosi 2003 | N | Y | U | Y | N | N | U | Y | 3 |
| Adegun 2017 | Y | Y | U | U | N | Y | Y | Y | 5 |
| Wang 2018 | Y | N | Y | Y | Y | Y | Y | Y | 7 |
| Li 2005 | N | Y | U | Y | Y | Y | U | Y | 5 |
| Naya 2008 | N | Y | U | Y | N | N | U | Y | 3 |
| Zhang 2016 | Y | N | Y | Y | N | N | Y | Y | 5 |
| Doğan 2015 | Y | Y | Y | Y | N | N | Y | Y | 6 |
| Mo 2014 | N | Y | Y | Y | N | N | Y | Y | 5 |
| Sonmez 2011 | Y | U | Y | Y | N | Y | Y | Y | 6 |
| Rhoden 2008 | Y | Y | Y | Y | Y | Y | Y | Y | 8 |
| Fwu 2014 | U | U | Y | Y | N | N | Y | Y | 4 |
| Gao 2015 | N | Y | Y | Y | Y | Y | Y | Y | 7 |
| Macnab 2019 | U | U | Y | Y | N | N | Y | Y | 4 |
| Note: Y-Yes, N-No, U-Unclear, NA-Not applicable | | | | | | | | | |
| ① = Were the criteria for inclusion in the sample clearly defined? | | | | | | | | | |
| ② = Were the study subjects and the setting described in detail? | | | | | | | | | |
| ③ = Was the exposure measured in a valid and reliable way? | | | | | | | | | |
| ④ = Were objective, standard criteria used for measurement of the condition? | | | | | | | | | |
| ⑤ = Were confounding factors identified? | | | | | | | | | |
| ⑥ = Were strategies to deal with confounding factors stated? | | | | | | | | | |
| ⑦ = Were the outcomes measured in a valid and reliable way? | | | | | | | | | |
| ⑧ = Was appropriate statistical analysis used? | | | | | | | | | |


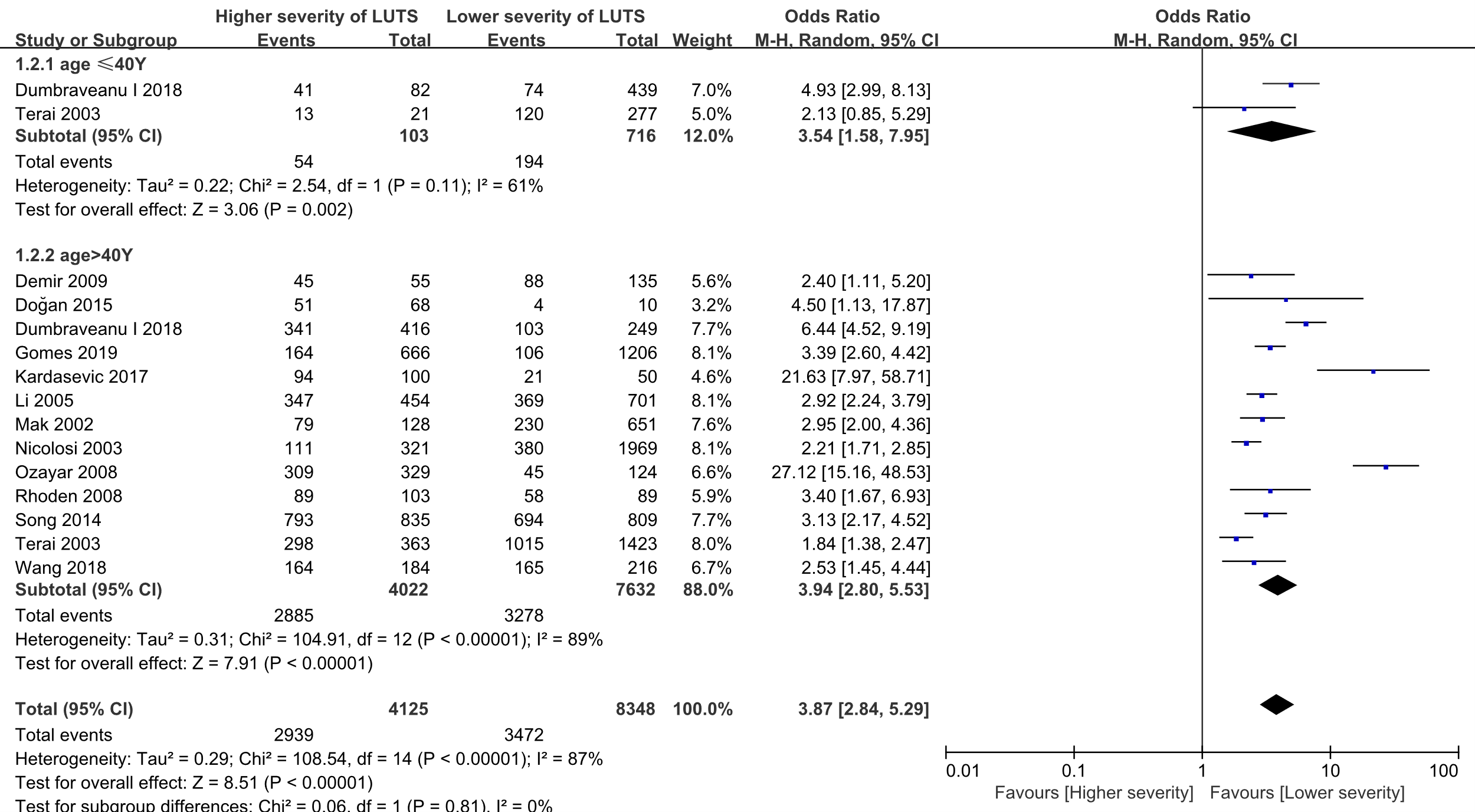


Fig S1 Forest plot showing subgroup analysis results of the association between LUTS severity and sexual dysfunction prevalence according to different ages.

LUTS = Lower Urinary Tract Symptoms; CI = Confidence Interval;


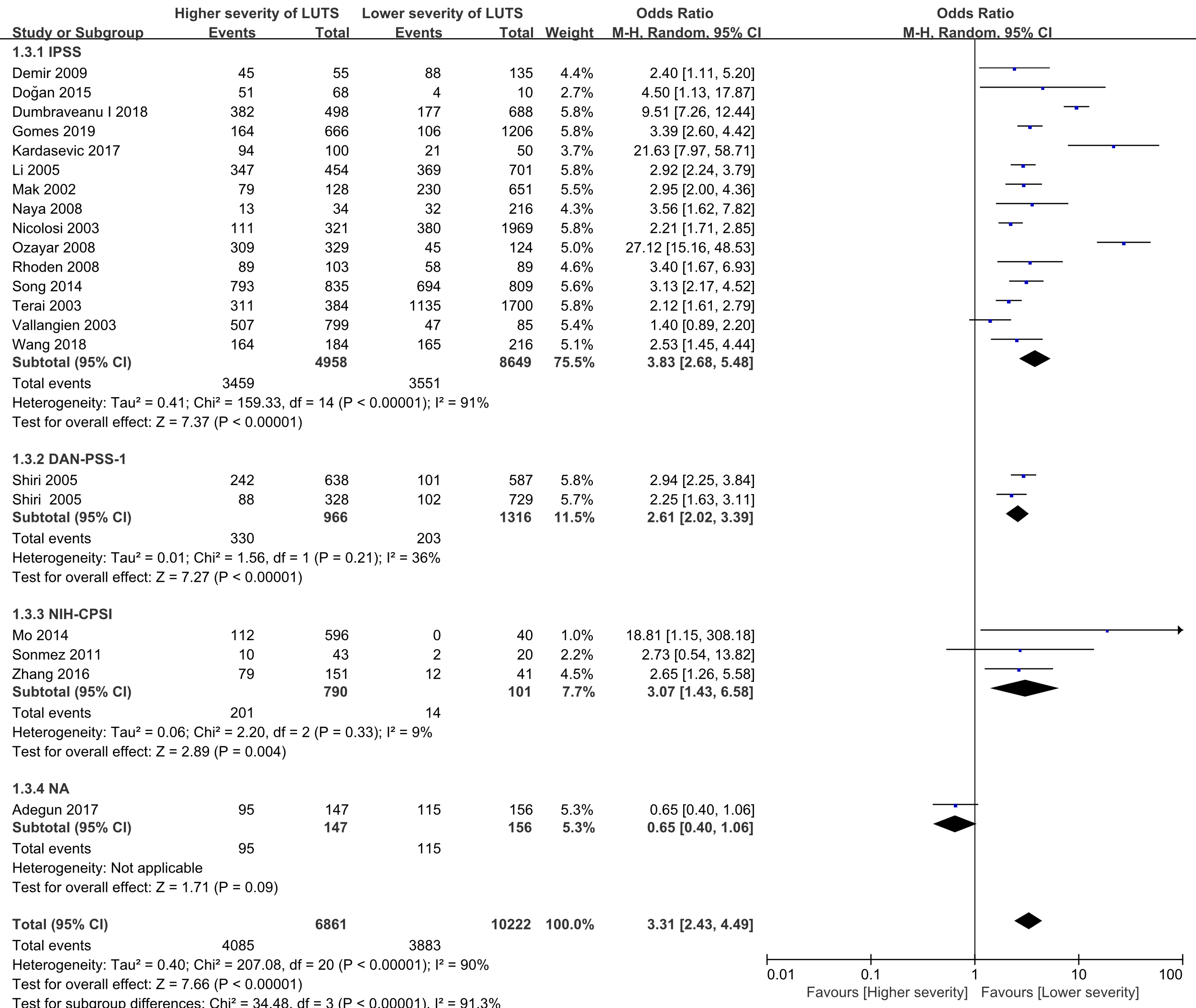


Fig S2 Forest plot showing subgroup analysis results of the association between LUTS severity and sexual dysfunction prevalence according to different LUTS assessment tools.

LUTS = Lower Urinary Tract Symptoms; CI = Confidence Interval;


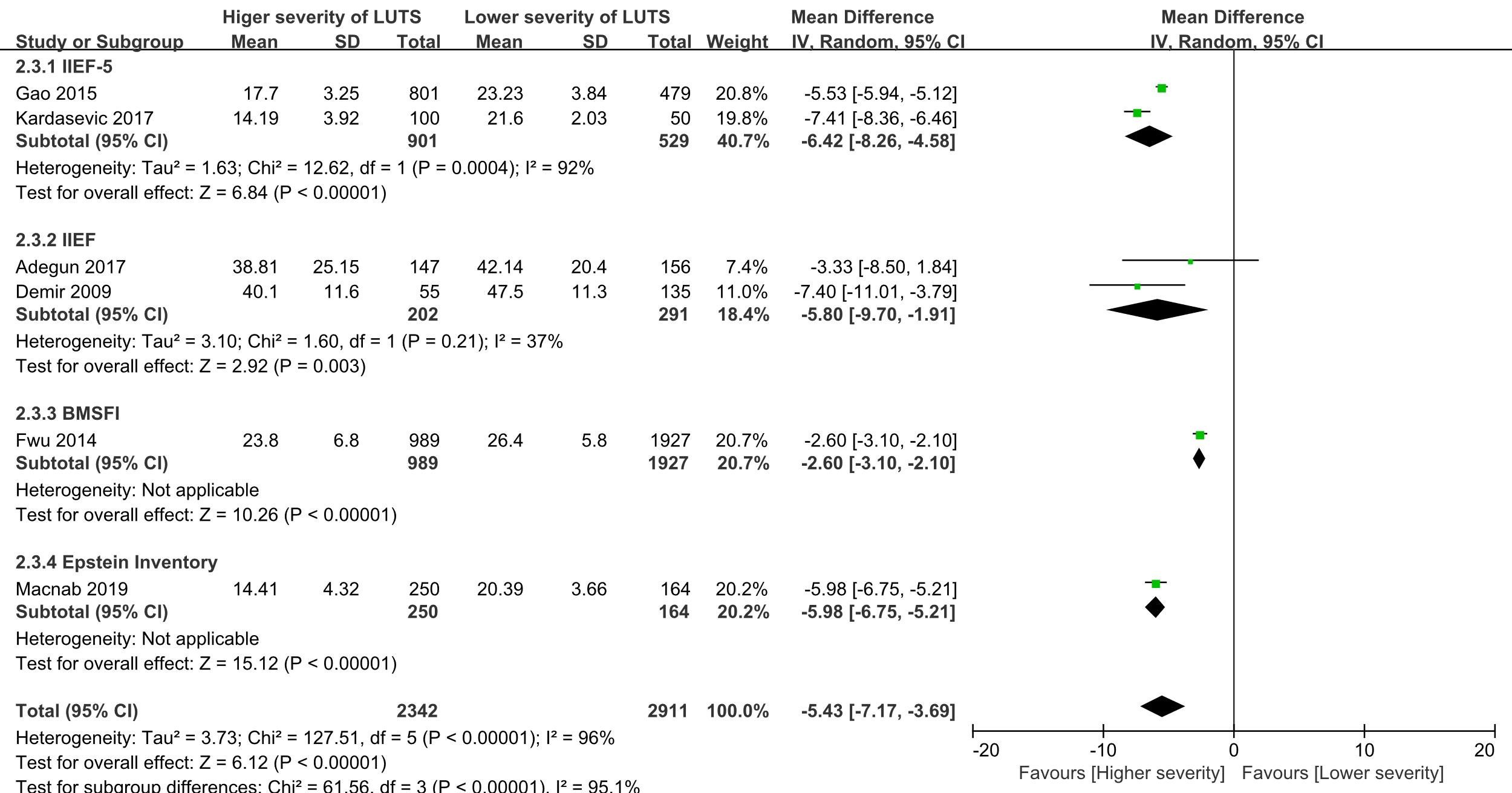


Fig S3 Forest plot showing subgroup analysis results of the association between LUTS severity and scores of sexual dysfunction assessment tools according to different sexual dysfunction assessment tools.

LUTS = Lower Urinary Tract Symptoms; CI = Confidence Interval; SD = Standard Difference
